# Perspective: Restorative reflective supervision: introducing *Pause and Reflect* a practical tool for supporting nurse well-being, learning, and retention

**DOI:** 10.1177/17449871251380950

**Published:** 2025-10-21

**Authors:** Sarah Bekaert

**Affiliations:** Oxford School of Nursing and Midwifery, Oxford Brookes University, Oxford, UK

This short perspective piece introduces *Pause and Reflect*, a practical tool for guided Restorative Reflective Supervision. It describes current workforce challenges that restorative reflective practice seeks to mitigate, the theoretical foundations of restorative reflective supervision, and the policy context in which it operates. The paper describes *Pause and Reflect*, a novel and structured model for guided restorative reflective supervision. It outlines the core delivery approach, which integrates the reflective elements within the restorative framework. Particular emphasis is placed on its practical implementation.

## Current workforce challenges

In today’s increasingly complex and emotionally demanding healthcare environments, supporting the well-being of nurses and other healthcare professionals is essential. A growing body of evidence highlights the serious impact of burnout on the nursing workforce and on the quality of patient care. For instance, Jun et al. (2021) identified nurse burnout as an occupational hazard strongly associated with poorer safety outcomes, reduced quality of care, lower patient satisfaction and diminished organisational commitment and productivity. Kurtzman et al. (2022) found that pre-existing staffing shortages were severely exacerbated by the COVID-19 pandemic, reporting increased intentions to leave the profession, severe emotional exhaustion, depersonalisation, and a sense of reduced personal accomplishment among nurses. Structural risk factors included inadequate resources, lack of social support, and unsustainable workloads – all linked to rising levels of burnout and staff turnover.

More recently, Powell et al. (2024) conducted a qualitative study capturing the lived experiences of nurses during and after the pandemic. Key themes included recurring waves of compassion fatigue, moral injury, secondary trauma, and chronic burnout. Participants described ongoing distress and unresolved trauma, highlighting the urgent need for sustained, systemic support for the profession. The initial study exploring the usability and feasibility of the *Pause and Reflect* guided restorative reflective supervision resource with school nursing teams used the Professional Quality of Life (ProQoL) Scale (Stamm, 2010) to measure compassion fatigue, burnout and secondary trauma. Findings indicated that the school nursing workforce was on the brink of burnout, underscoring the high emotional toll of frontline public health nursing roles.

## What is restorative supervision?

Restorative supervision is a well-established approach shown to help reduce stress and burnout among nurses and midwives (Wallbank and Woods, 2012). Guided supervision and peer support are also increasingly recognised as effective ways to sustain professionals facing challenging workloads (see, e.g. literature reviews by Tulleners et al., 2022; Papathanasiou et al., 2023). The Restorative Reflective Supervision model, *Pause and Reflect*, combines these three elements: restorative aims, peer support, and a structured guided approach.

Traditional clinical and safeguarding supervision focus primarily on case-related issues, whereas restorative supervision offers a more person-centred approach for the practitioner. It recognises the emotional impact of clinical work and provides protected time for reflection and emotional processing. Developed in the United Kingdom (UK) in response to rising levels of stress, burnout, and staff attrition; restorative supervision aims to build resilience, encourage reflective practice, and sustain a healthy, motivated workforce (Wallbank and Woods, 2012). Where clinical and safeguarding models emphasise reflection, learning, and support in relation to specific cases, restorative supervision shifts the focus to the professional rather than the case itself (Butterworth, 2022). Restorative supervision supports practitioners in managing stress, anxiety, and burnout associated with complex caseloads and emotionally demanding roles. The process creates a safe, confidential space where feelings, experiences, and challenges can be explored without judgement - fostering trust and psychological safety.

Facilitated reflection enables practitioners to examine their own emotions, reactions and experiences, promoting deeper self-awareness and insight into their practice. This emotional processing strengthens the capacity to cope with difficult situations and maintain resilience. By prioritising well-being, restorative supervision can enhance job satisfaction, motivation and retention rates (Scanlan and Hart, 2024). Evidence shows it can increase compassion satisfaction and reduce burnout and stress by up to 40% and has been linked to improved staff retention and reduced sickness absence in midwifery services (Wallbank and Woods, 2012).

## The context for restorative supervision

Restorative supervision was highlighted in the government’s response to the Francis Report (Department of Health, 2013) as an example of how to promote a compassionate National Health Service (NHS) following an inquiry into failures in a specific hospital trust in England. In the post-pandemic context, there have been renewed calls for its broader implementation to address ongoing workforce challenges (Darzi, 2024).

The Wallbank model of Restorative Clinical Supervision, created by Dr. Sonya Wallbank in 2007, was initially designed to support healthcare professionals working in highly emotional contexts; for example, midwives caring for families after miscarriage, stillbirth, neonatal death, or trauma-related cases. Professional Midwifery Advocates (PMAs) were introduced around 2017 and formed the precursor to the Professional Nurse Advocate (PNA) role. PNAs are specially trained registered nurses, who provide restorative clinical supervision while advocating for staff well-being, professional growth and quality improvement across NHS services in England. The national PNA programme, launched in 2021 by NHS England, was developed to reduce burnout and moral injury, retain staff, and improve both patient safety and quality of care (NHS England, 2021).

Both PMAs and PNAs work within the Advocating and Educating for Quality Improvement (A-EQUIP) model (NHS England, 2021). A-EQUIP expands Proctor’s (1986) classical three-function clinical supervision framework developed within social work: normative, formative, and restorative; by adding a fourth dimension – personal action for quality improvement. The *normative* function addresses monitoring, evaluation and quality control, supporting accountability and professional standards. The *formative* function focuses on education, development and reflective learning, including skills enhancement and preparation for revalidation. The *restorative* function provides a safe space to explore emotional impact, reduce burnout and build resilience. Therefore, restorative supervision is a central element of the A-EQUIP model with a specific focus on staff well-being. The *personal action for quality improvement* dimension encourages practitioners to apply insights from supervision to actively enhance service quality.

## *Pause and Reflect*: a structured model for guided restorative reflective supervision

The need for a structured model for restorative supervision emerged from focus groups with school nurses (Bekaert et al., 2023). These discussions highlighted the emotional demands of the role, the risk of burnout, and the value of having a safe, guided space for reflection – and the need for structural change. This insight led to the co-design and national validation of a resource tailored to their needs. The resulting restorative reflective model, *Pause and Reflect*, has transferability across healthcare settings and professions. It provides a clear, practical framework for those delivering restorative supervision, ensuring consistency, safeguarding of psychological safety, and promotion of resilience among practitioners.

The model builds the reflective process into the restorative focus, supporting a guided transition from emotional expression to critical thinking and action. Reflection is central to nursing, drawing on interdisciplinary knowledge and developed through experience. Bulman and Schutz (2013) described reflective practice as the organising theory of nursing. Further, the NMC (2018) identified reflective practice as vital for maintaining standards, enhancing care and supporting professional growth. By embedding the reflective process within restorative supervision, *Pause and Reflect* helps practitioners work through emotions, gain insights into their practice, and apply learning to real-world contexts. This approach not only strengthens individual resilience but also fosters professional confidence, improves decision-making and contributes to safer, more compassionate care.

The reflective element of the resource draws on Gibbs’ (1988) six-stage reflective cycle: description, feelings, evaluation, analysis, conclusion, and action planning. In the *Pause and Reflect* model, the restorative element is integrated into the first two stages: exploring what happened or is happening, and how it feels; before progressing to consideration of what needs to change and the practical steps to achieve this. Linking restorative support with structured reflection is a novel feature of the resource. Goals developed through this process should be specific, measurable, achievable, relevant, time-bound (SMART; Doran, 1981) and focus on systemic improvements that enhance working conditions and address workforce challenges in a practical, sustainable way.

## Delivery process

The following section presents a step-by-step guide for delivering the *Pause and Reflect* model in practice.

### Step 1: Setting up the session

The session should begin with a brief explanation of restorative reflective supervision, including its role and focus. Ground rules should be agreed upon within the group. The facilitator should highlight available sources of support outside the session and clearly outline their safeguarding responsibilities.

### Step 2: Completing the ProQoL questionnaire

It is recommended that each participant complete an anonymous ProQoL questionnaire. The ProQoL framework (Stamm, 2010) is widely used in helping professions to assess three key areas: compassion satisfaction – the sense of fulfilment from helping others; burnout – emotional exhaustion and a reduced sense of accomplishment and secondary traumatic stress (or compassion fatigue) – stress resulting from exposure to others’ trauma. Aggregated results provide a quantitative measure of staff or team well-being, useful for advocacy and supporting requests for systemic changes to benefit the workforce.

### Step 3: The guided restorative reflection process

The third step is the guided restorative reflection process, which comprises three key stages:

- Description of events and personal impact: The facilitator invites participants to share experiences that affected them professionally, such as interprofessional interactions or patient-related events.
- Critical discussion: The facilitator explores participants’ feelings, highlights positive actions, and guides discussion on constructive alternative approaches.
- Planning for practice: Participants consider necessary changes and develop SMART goals to implement improvements in practice.

### Step 4: Grounding and closure

The next step is grounding and closure, where the facilitator brings the discussion and planning to a close. Participants are reminded of available sources of support outside the restorative reflective sessions. The session should end with a lighter, informal group discussion, such as local events or general well-being topics, to help participants transition back to their day. Providing refreshments, such as tea and cake, can further support a relaxed and positive closure, reinforcing a sense of connection and well-being within the group.

### Step 5: Evaluation

Finally, it can be helpful to ask participants to complete an evaluation questionnaire. A questionnaire has been adapted from an existing validated clinical supervision evaluation tool (Horton et al., 2008) and further validated with the school nursing teams from the initial study [available on request from the author]. It collects feedback using Likert-scale responses on participants’ understanding of the purpose of restorative reflective supervision, as well as their perceptions of safety, trust and confidence in the process. The questionnaire also assesses the immediate impact of the session on self-awareness, coping, and professional confidence. Qualitative feedback is gathered through an open-ended question inviting any other comments or observations. The results provide useful evidence of the value of restorative reflective supervision to managers and stakeholders, supporting workforce advocacy and decisions about ongoing implementation.

When restorative reflective supervision is delivered on a regular basis, previously established goals can be reviewed and updated to reflect progress or emerging needs. Repeated administration of the ProQoL questionnaire allows for the monitoring of changes over time in secondary traumatic stress, burnout, and compassion fatigue. Additionally, the collection of evaluative feedback from participants provides actionable insights that can be used to refine and enhance the delivery of the sessions, ensuring they remain responsive, effective and aligned with the needs of the workforce.

## Conclusion

*Pause and Reflect* offers a structured, practical tool for delivering guided restorative reflective supervision, designed to support nurse well-being, learning and retention. Its novel integration of restorative support with structured reflective practice provides a clear framework for facilitators while promoting emotional processing, critical reflection and actionable improvements in practice. By embedding evaluation, through tools such as ProQoL and session feedback, *Pause and Reflect* generates evidence of both individual and team impact, helping managers and stakeholders recognise its value, and supporting advocacy for systemic change. This combination of practical guidance, innovative design, and evaluative capacity makes *Pause and Reflect* a timely and pragmatic approach to sustaining a resilient, motivated healthcare workforce.
